# A Delphi panel to build consensus on assessing disease severity and disease progression in adult patients with hypophosphatasia in the United States

**DOI:** 10.1007/s40618-023-02256-4

**Published:** 2024-01-18

**Authors:** K. M. Dahir, E. T. Rush, S. Diaz-Mendoza, P. S. Kishnani

**Affiliations:** 1 Division of Endocrinology and Metabolism, Vanderbilt University Medical Center, 1215 21st Ave S Suite 8210, Nashville, TN 37232 USA; 2 Division of Clinical Genetics, Children’s Mercy Kansas City, 2401 Gillham Rd, Kansas City, MO 64108 USA; 3 Department of Pediatrics, University of Missouri-Kansas City School of Medicine, Kansas City, MO USA; 4 Department of Internal Medicine, University of Kansas School of Medicine, Kansas City, KS USA; 5 OPEN Health Group, Marlow, UK; 6 Division of Medical Genetics, Department of Pediatrics, Duke University School of Medicine, Durham, NC USA

**Keywords:** Hypophosphatasia, Delphi, Consensus

## Abstract

**Background:**

Hypophosphatasia (HPP) is an inborn error of metabolism with a variable presentation. We conducted a modified Delphi panel to obtain expert consensus on knowledge gaps regarding disease severity and progression in adult patients with HPP.

**Methods:**

Healthcare professionals (HCPs) with experience managing adult patients with HPP were recruited to participate in a 3-round Delphi panel (round 1: paper survey and 1:1 interview; rounds 2–3: email survey). Panelists rated the extent of their agreement with statements about disease severity and progression in adult patients with HPP. Consensus was defined as ≥ 80% agreement.

**Results:**

Ten HCPs completed round 1; nine completed rounds 2 and 3. Consensus was reached on 46/120 statements derived from steering committee input. Disease severity markers in adult patients with HPP can be bone-related (recurrent/poorly healing fractures, pseudo-fractures, metatarsal fractures, osteomalacia) or involve dentition or physiologic/functional manifestations (use of mobility devices/home modifications, abnormal gait, pain). Disease progression markers can include recurrent/poorly healing low-trauma fractures, development of ectopic calcifications, and/or impairment of functional activity. Panelists supported the development of a tool to help assess disease severity in the clinic and track changes in severity over time. Panelists also highlighted the role of a multidisciplinary team, centers with expertise, and the need to refer patients when disease severity is not clear.

**Conclusions:**

These statements regarding disease severity, progression, and assessment methods address some knowledge gaps in adult patients with HPP and may be helpful for treating HCPs, although the small sample size affects the ability to generalize the healthcare provider experience.

**Supplementary Information:**

The online version contains supplementary material available at 10.1007/s40618-023-02256-4.

## Background

Hypophosphatasia (HPP) is a rare, inherited, metabolic disease caused by loss of function variants in the *ALPL* gene resulting in deficiency of tissue-nonspecific alkaline phosphatase (TNSALP) [1, 2]. HPP has a highly variable and progressive clinical presentation [3–10]. Patients with HPP often have defective bone mineralization that can lead to an increased propensity of fracture and poor mineralization of teeth or defects in acellular cementum that can lead to early tooth loss. In the HIPS and HOST surveys, the most commonly reported signs and symptoms in adult patients with HPP were pain, fractures, muscle weakness, and abnormal gait [11]. Morbidities may develop over a patient’s lifetime to include recurrent or poorly healing bone fractures, rheumatologic manifestations, orthopedic surgeries, and dental manifestations [5, 12]. HPP causes impaired mobility and impacts patients’ functional status [11–13] and has a profound negative impact on patients’ health-related quality of life [11, 14].

Diagnosis and assessment of disease severity in HPP are made by an aggregate of findings (e.g., biochemistry, musculoskeletal abnormalities, and molecular testing) and not by genotype alone [15]. Diagnosis is typically based on low alkaline phosphatase (ALP) activity (age and sex-specific) as well as genotype, once other causes of low ALP activity are excluded. Measurement of ALP substrates such as pyridoxal-5-phosphate (PLP) can also assist with diagnosis: the combination of low ALP activity and elevated PLP levels is suggestive of HPP and can be used to differentiate it from secondary reasons for low ALP activity [16–18].

Historically, disease severity has been observed to reflect patient age at first sign/symptom onset, and HPP has been categorized into perinatal, prenatal benign, infantile, childhood (differentiated into mild versus severe forms [19]), and adult HPP [2, 18, 20]. These age-based categories have been important to our understanding of HPP, and the categorizations have been useful to date by providing a clinical construct for the diagnosis and management of HPP. However, HPP has one of the broadest ranges of severity among all inherited skeletal diseases, and there is considerable variability in presentation within and across age groups and clinical subgroups and even within families [2, 18, 20, 21]. Patients can also present with odontohypophosphatasia, which is disease limited to the teeth that can cause premature tooth loss before 5 years of age or other dental complications at any age [18]. However, care should be taken in the diagnosis of odontohypophosphatasia, as patients may develop additional signs and symptoms of more involved disease later on [22]. HPP-related clinical manifestations and events can accumulate and/or change over time (e.g., evolution from dental abnormalities to skeletal manifestations or from ambulatory to nonambulatory later in life), so it is becoming clear that the current categorizations describe a disease continuum, rather than separate disease forms [22]. This is similar to X-linked hypophosphatemia (XLH), which was historically considered a pediatric disease. However, more recently, adults with XLH have been reported to show progressive consequences of childhood disease with signs (e.g., short stature, lower limb deformity) and ongoing symptoms (e.g., impaired muscle function, osteomalacia, osteoarthritis) [23]. Therefore, it is important to evolve the nosology of HPP and think of it as a continuum as we continue to learn more about the disease.

Gaps in understanding the evolution of HPP still exist, including disease severity and disease progression, which are confounded by the fact that the disease presents with substantial heterogeneous manifestations. There is no standardized clinical definition of disease severity. Some physicians may use the Six-Minute Walking Test (6MWT) or Timed Up and Go (TUG) Test to assess and/or monitor disease progression in patients with HPP. In the 6MWT, which is validated in patients with HPP, patients are instructed to walk as far as possible in 60-m laps along the length of a hallway for 6 min, and the total number of meters walked (i.e., Six-Minute Walking Distance [6MWD]) is recorded [24, 25]. The TUG Test, which has not yet been validated in patients with HPP, measures the time it takes a patient to stand from sitting, walk 3 m, turn, walk 3 m back, and sit again [26]. However, these tests may not be used or readily available at all centers or physicians treating HPP. Disease severity and rate of disease progression may be difficult to define in rare diseases such as HPP, and these aspects of chronic disease are important gaps in our current knowledge of HPP. The ability of healthcare providers (HCPs), patients, and caregivers to recognize disease severity and/or progression is critical to the appropriate management of patients with HPP.

A Delphi panel allows for anonymous, iterative collection and statistical aggregation of informed judgements from experts; it is characterized by repeated rounds of controlled feedback until consensus is achieved [27]. The Delphi method is widely used in healthcare research and is proven to be a rigorous and feasible way to obtain consensus [28, 29]. A modified Delphi panel was conducted to obtain expert consensus on (1) how to describe and assess disease severity in adult patients with HPP and (2) how to describe and monitor disease progression in adult patients with HPP in a clinical practice setting.

## Methods

### Study design

**Fig. 1:**
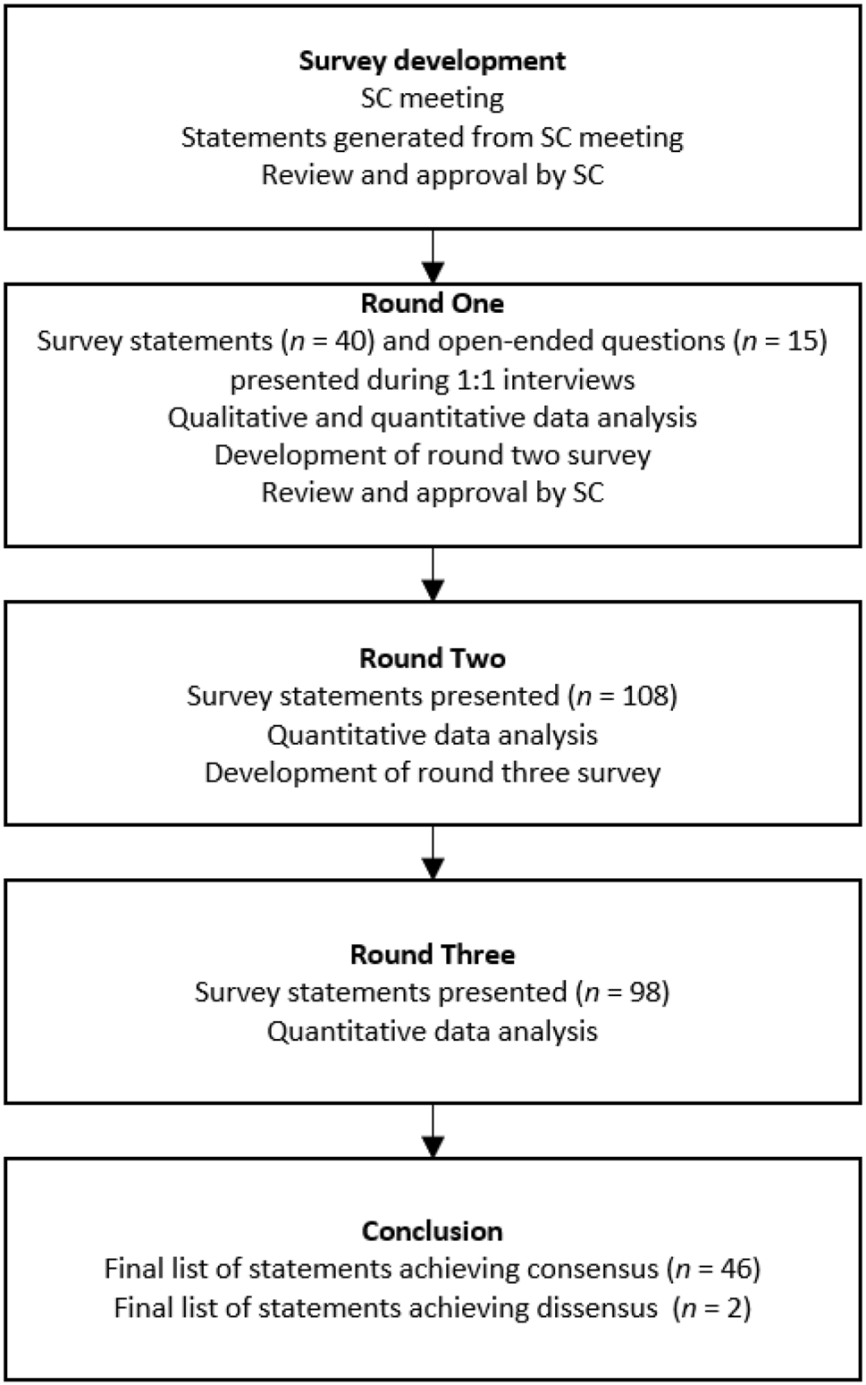
Modified Delphi framework. *SC* steering committee

To meet the objectives, a modified three-round Delphi panel was conducted. Delphi panels have previously been used to better understand disease severity [30] and progression [31]. Modifications made to the Delphi panel methodology included a single 1:1 interview round and two survey rounds (Fig. 1). The first round 1 survey was also developed based on a discussion with the steering committee (SC), rather than from an initial open-ended round of statements (classical Delphi panel) [32]. Three HCPs (KMD, ETR, and PSK) with expertise in treating adults who live with HPP formed the Delphi Panel SC, providing input to the study design, potential panelists, and survey development. Per governance arrangements for research ethics committees, review is not required for research involving healthcare professionals recruited as research participants by virtue of their professional role [33], so institutional ethics committee approval was not deemed necessary.

### Panelist selection

The SC invited 31 US-based HCPs to participate in the Delphi panel, in an attempt to reach a target sample size of 12 experts (as per the recommended sample size of 5–20 individuals [34]). A larger sample would not have been achievable given the pool of HCPs with expertise in managing adult patients with HPP is limited because of the rare nature of the disease. The sample was limited to US-based HCPs due to differences in clinical practice between countries. Invited HCPs were required to manage adult patients with HPP in their regular practice.

### Preparation

An SC meeting was held in November 2020 to discuss the research objectives and develop initial survey statements. The SC outlined the Delphi panel statement framework to address two objectives: (1) how to describe and assess disease severity in adult patients with HPP and (2) how to describe and monitor disease progression in adult patients with HPP in the clinical practice setting. The SC discussion was used to develop the first survey draft, and statements/open-ended questions were drafted for each domain. The first survey draft was then distributed to the SC for review and approval. Statements/open-ended questions regarding objective 1 (disease severity) were further divided into four sections (Table 1). The SC decided not to present specific definitions of disease severity, given the complexity of the disease and broad spectrum of signs and symptoms. Instead, the aim of this study was to provide a framework of elements for clinicians to consider when assessing disease severity for individual adult patients.

**Table 1:** Statement framework

Domain	Sub-domain
Disease severity	Feasible and appropriate methods to assess disease severity in adult patients with HPP
	Markers of disease severity in adult patients with HPP
	Nuances of disease severity assessment in adult patients with HPP
	Challenges surrounding tests conducted in clinic to assess disease severity in adult patients with HPP
Disease progression	No sub-domains

*HPP* hypophosphatasia

### Procedure

The Delphi panel was conducted between November 2020 and July 2021. Potential panelists were invited via email to participate in the Delphi panel; the email detailed information about the study, its objectives, and what participation entailed. Written consent was obtained from willing participants, whose round 1 interviews were then scheduled. Round 2 and 3 surveys were emailed to panelists, who were typically given 14 days to complete and return their responses (reminders were sent at regular intervals by project manager or Delphi SC member).

### Survey development

Statements addressing the objectives were developed and shared with the panelists across three rounds. During each round, panelists were asked to rate the extent to which they agreed with each statement using either Likert scales or binary responses. In round 1, all statements were presented with a 5-point Likert response scale (1 = completely disagree, 2 = not agree [renamed disagree in rounds 2 and 3], 3 = neutral, 4 = agree, 5 = completely agree). Open-ended questions were only asked during round 1 to generate further statements for round 2. Areas for comments under each statement were also incorporated, allowing panelists to provide additional qualitative insights. After the first round, 3-point Likert scale (1 = disagree, 2 = neutral, 3 = agree) or binary response options (disagree, agree) were selected by the researchers and approved by the SC depending on the percentage frequencies a statement achieved in the prior round, as per the analysis rules.

### First round

The round 1 (November 2020–March 2021) survey was completed by panelists during a 1:1 audio-recorded teleconference interview with a researcher, to facilitate discussion of the statements. Panelists were provided a structured list of 40 statements and 15 open-ended questions to collect both quantitative and qualitative data. Panelists’ qualitative data were used to generate further statements for the round 2 survey (Fig. 1).

### Second and third round

According to the modified Delphi methodology, all open-ended questions and the 12 statements that did not achieve the minimum response threshold (41%) during round 1 were removed from the survey. Eighty new statements were generated following analysis of panelists’ qualitative responses to the open-ended questions and their comments on preexisting statements, resulting in a 108-item round 2 survey. The round 2 survey was customized for each panelist, presenting panelists’ individual responses and the group mode, mean, and interquartile range (IQR) for statements brought forward from round 1. Round 2 was conducted between May and June 2021.

Following quantitative analysis of the round 2 survey data, ten statements were removed (eight achieving consensus, two not achieving the minimum response threshold) as per the analysis rules, leaving 98 items in the round 3 survey (Fig. 1). Similar to round 2, individual and group responses were reported to panelists in the round 3 survey (sent to panelists in July 2021).

### Data analysis and definition of consensus

Qualitative comments and answers from the panelists’ round 1 interviews were reviewed and addressed either to refine existing statements or to create new statements for the round 2 survey. After each round, quantitative survey responses were extracted for each statement into a Microsoft Excel database and were assigned a score/code (i.e., 1–5, 1–3, or AG/DG) corresponding to each Likert/binary response scale. The IQR was calculated and used to summarize the extent of the spread of the data. Central tendencies (mean, median, and mode) were calculated to present the group’s responses back to panelists, and percentage response frequencies for each statement were calculated to determine whether consensus had been achieved. The consensus definition was determined a priori with the SC and was later refined and standardized into the following set of analysis rules (Table 2).

**Table 2:** Analysis rules

Rule 1: Questions that show variable response patterns (≤ 40%) spread across response options in a non-skewed way will be removed
Rule 2: Questions with responses between 41 and 79% will be re-asked with three response options: disagree, neutral, and agree
Rule 3: Questions that showed skewed response pattern, with the majority of responses (≥ 80%) spread across 5 or 3 options, will be summed and asked back with a binary response option: agree or disagree
Rule 4: Binary questions that showed a response pattern of ≥ 80% agreement will be considered consensus
Rule 5: Three-point Likert scale questions in the second round with responses between 41 and 79% will be re-asked on a three-point Likert scale in Round 3
Rule 6: Three-point Likert scale questions in the third round with ≥ 80% of a response option will be considered as consensus

## Results

### Participation in the survey

Out of the 31 HCPs invited, 12 accepted the invitation to participate but only ten scheduled Round 1 interviews. Ten HCPs participated in Round 1 and nine completed Rounds 2 and 3. All panelists had experience in managing adult patients with HPP. In addition, the nine panelists belonged to one, or a combination of, the following specialties: (1) pediatrics, (2) pediatric endocrinology, (3) internal medicine, (4) clinical molecular/clinical genetics, and (5) adult endocrinology.

### Overview of results

**Fig. 2:**
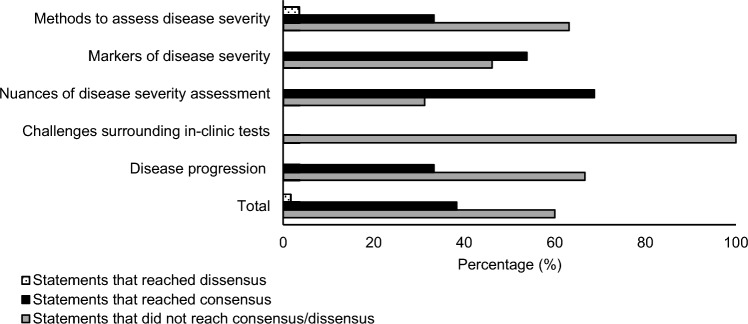
Proportion of statements that achieved consensus, dissensus, and that did not reach either

Overall, 46 (38.33%) of the 120 statements presented to panelists across all three Delphi rounds achieved consensus, and two (1.68%) statements achieved dissensus (Fig. 2). Of the 92 statements presented in the disease severity domain, 37 (40.22%) achieved consensus and two (2.27%) achieved dissensus. The sub-domains “nuances of disease severity assessment” (68.75%) and “markers of disease severity” (58.33%) contained the highest proportion of statements achieving consensus, and “methods to assess disease severity” contained the highest proportion of statements achieving dissensus (3.51%). No statements from the “challenges surrounding in-clinic tests” sub-domain achieved consensus/dissensus. From the 27 statements presented in the disease progression domain, nine (33.33%) achieved consensus.

### Objective 1: Disease severity

#### Section 1: Methods to assess disease severity

Within Sect. 1, 19 (33.3%) of the 57 presented statements achieved consensus, and two (3.51%) achieved dissensus (Table 3). Panelists agreed on eight feasible and appropriate methods to assess the disease severity of adult patients living with HPP in clinic: (1) Clinical consultations with the patient, (2) assessing quality of life via patient’s history and self-report, (3) assessments using pain scales, (4) assessing the types of pain medications the patient is taking, as prescribed by a specialist, (5) Assessing the patient’s medical history, (6) checking for nephrocalcinosis, (7) assessing patient’s gait, and (8) examining patient’s musculoskeletal condition (Supplemental Table 1, Additional File 1). Dissensus was achieved for two statements in this section: Panelists disagreed that (1) as a treating physician, they would conduct functional assessment in clinic of a treatment-naïve adult patient every 1 to 3 months or (2) refer the patient to a physical therapist/occupational therapist to conduct functional assessments on a 1-to-3-month timeframe.

**Table 3:** Top consensus and dissensus areas among panelists

Top consensus (100% agree)	Top dissensus (100% disagree)
**Feasible and appropriate methods to measure disease severity**	
Clinical consultations Assessing the patient’s medical history Examining patient’s musculoskeletal condition Role of physical therapist/occupational therapist to conduct: assessments of functional impairment using clinical scales, assessments of muscle fatigue, the Timed Up and Go Test, and the Six-Minute Walk Test^a^ Receiving objective data from physical therapists/occupational therapists (who are knowledgeable about HPP), providing valuable information with which to compare adult patients with HPP with controls on age- and sex-adjusted variables	A timeframe of every 1–3 months to refer patients to physical/occupational therapists to conduct functional assessments
**Markers of disease severity**	
Primary and secondary dentition manifestations Mobility devices and home modifications Abnormal gait (worsens over time/does not improve) Positive correlation between the amount of pain reported and disease severity	NA
**Nuances of disease severity assessment**	
Referral to centers with expertise Contextualizing evidence from X-ray and advanced radiologic imaging when assessing disease severity Assessment in context of possible comorbidities Consulting other colleagues and literature Additional work-up	NA
**Challenges surrounding in-clinic tests**	
NA	NA
**Disease Progression**	
In adult patients with HPP – Fractures which heal poorly/take a long time to heal – Recurrent fractures – Evidence of low-trauma fractures – Impairment of daily functional activity In adult patients with HPP who present in a wheelchair OR who have functional impairment – New fractures After seeing an adult patient with HPP who has begun enzyme replacement therapy every 3 months for the first year, you would ask to see that patient every 6 months to a year, to monitor disease progression	NA

*HPP* hypophosphatasia, *NA* not applicable

^a^For additional information on the Timed Up and Go Test and Six-Minute Walk Test, please see Supplemental Table 2, Additional File 1

Statements that did not achieve consensus or dissensus as feasible and appropriate methods for the treating physician to assess disease severity in adult patients with HPP were related to using clinical scales to assess well-being (e.g., The 36-Item Short Form Survey) or quality of life (e.g., LEFS, WOMAC, PROMIS); using clinical scales to assess functional impairment (e.g., PROMIS, GMFM-88, and GSGC) or fatigue (e.g., the PROMIS Fatigue-Short Form, the Fatigue Severity Scale); DXA measurements (performing a DXA scan at sites such as the hip [femoral neck, total femur] and spine); and functional testing (conducting the Five Times Sit-to-Stand Test; conducting the 6MWT; conducting the TUG Test; and the 6MWT conducted by a pulmonary laboratory in lieu of the treating physician). For additional information on the TUG Test and 6MWT, please see Supplemental Table 2, Additional File 1.

Regarding the assessment/examination of a patient’s musculoskeletal condition, four methods reached consensus: (1) assessing the patient’s medical history, (2) general ability to walk, and (3) gait, and (4) performing X-rays to assess for fractures. However, panelists did not agree that conducting a (1) Sit-to-Stand Test or (2) 6MWT is a feasible or appropriate method to examine patients’ musculoskeletal condition.

Additionally, the panel reached consensus that evidence from X-ray and advanced radiologic imaging should be contextualized as part of the entire clinical picture/assessment/formulation when assessing disease severity.

The panel agreed that a physical or occupational therapist (in lieu of the treating physician) may be able to assess disease severity through assessments of functional impairment using clinical scales (e.g., PROMIS, GMFM-88, and GSGC), assessments of muscle fatigue, quantifying the level of weakness displayed by the patient through objective methods such as hand grip strength or leg extensions, and by using assessment tools such as the Five Times Sit-to-Stand Test, 6MWT, or TUG Test.

#### Section 2: Markers of disease severity

Out of 12 markers of disease severity, seven (58.33%) achieved consensus, including (1) presence of fractures, pseudo-fractures, and metatarsal fractures which heal poorly or are recurrent; (2) fractures that occur spontaneously or through low-impact trauma; (3) primary (e.g., premature loss of teeth without root resorption, excess caries) and secondary (e.g., periodontal disease, excess caries, tooth loss) dentition manifestations in patients with a confirmed diagnosis of HPP; (4) use of mobility devices and home modifications because of HPP; (5) abnormal gait which worsens over time or does not improve; (6) a positive correlation between the amount of pain an adult patient with HPP reports and the severity of their disease; and (7) evidence of osteomalacia identified following a bone biopsy.

#### Section 3: Nuances of disease severity assessment

Within Sect. 3, 11 (68.75%) out of 16 statements reached consensus. Panelists recognized that disease severity may change over time, particularly in adult patients with HPP, and supported the development of a tool that would aid in the assessment of disease severity in the clinic setting, enabling them to track changes in severity over time. Although categorizations of disease severity in HPP do not yet exist, panelists agreed with the statement that it would be appropriate to categorize disease severity in adult patients with HPP into three categories—mild, moderate, and severe—in conjunction with the current nosology for HPP.

Additionally, panelists agreed that it is important to assess the disease severity of adult patients with HPP in the context of possible comorbidities present and that when assessing disease severity evidence from X-ray and advanced radiologic imaging, it should be contextualized as part of the entire clinical picture/assessment/formulation.

Consensus was also achieved on the role of specialist centers (i.e., centers with expertise) and the need to refer patients when disease severity is not clear. Moreover, panelists agreed that when assessing clinical findings or patient reported signs and symptoms that are not in keeping with their understanding of HPP, it is important to (1) consult the available literature; (2) consult with other clinicians who are knowledgeable about adult HPP; (3) consider additional work-up to confirm whether HPP is the cause of the patient’s signs or symptoms; and (4) consider referring the patient to a specialist (if you do not belong to that specialty).

#### Section 4: Challenges surrounding in-clinic tests

The panelists did not reach consensus on any statements regarding challenges surrounding tests conducted in clinic to assess disease severity in adult patients with HPP.

### Objective 2: Disease progression

Of the 27 statements on the topic of disease progression in adult patients with HPP, nine (33.33%) achieved consensus. The researchers suggested to differentiate the manifestations of disease progression between adult patients with HPP and a subgroup of these patients who are nonambulatory or who live with functional impairment. Panelists agreed that disease progression in adult patients with HPP can include any of the following manifestations: (1) evidence of fractures resulting from low trauma, (2) development of ectopic calcifications (either in eyes, kidneys, or joints), (3) recurrent fractures, (4) impairment of daily functional activity, and (5) fractures which heal poorly/take a long time to heal. Panelists agreed that disease progression in adult patients who present in a wheelchair or with functional impairment could reasonably include one or more of the following: (1) new fractures, (2) development of new calcifications, and (3) loss of ability to transfer from wheelchair to other areas (e.g., from wheelchair to their bed). Panelists also provided input on follow-up consultations for adult patients with HPP being treated with enzyme replacement therapy, recommending they be seen every 3 months for the first year and then every 6–12 months thereafter to monitor disease progression.

## Discussion

Using a modified Delphi panel, we aimed to obtain expert consensus among US HCPs on how to describe both disease severity and disease progression and how to monitor disease progression in adult patients with HPP. It is challenging to obtain consensus on a rare disease with a heterogeneous presentation of signs, symptoms, and severity/impact on activities of daily living. We identified 48 (46 consensus and 2 dissensus) statements that could aid in the development of consensus descriptions of disease severity and disease progression in adult patients with HPP.

Markers of disease severity to consider in adult patients with HPP include bone-related manifestations (e.g., recurrent or poorly healing fractures, pseudo-fractures, and metatarsal fractures; osteomalacia), dentition (primary and secondary), and physiologic/functional manifestations (e.g., use of mobility devices and home modifications, abnormal gait, pain). Historically, skeletal manifestations were considered the key signs of severe disease [2]. However, muscular and pain manifestations are now being increasingly recognized as contributing to disease severity [11, 35]. Gait, pain, and mobility device use could be a cluster to describe severe disease as these are often primary manifestations seen in clinical practice.

The panelists agreed that manifestations of disease progression in adult patients with HPP can include evidence of low-trauma, recurrent, or poorly healing fractures; development of ectopic calcifications; and impairment of daily functional activity. Manifestations of disease progression in adult patients with HPP who already present with functional impairment or in a wheelchair can include new fractures, new calcifications, and/or a loss of ability to transfer from wheelchair to other areas.

A goal of this Delphi panel was to gather input from panelists about monitoring adult patients with HPP for disease progression to fill this knowledge gap. The statements reaching consensus on disease progression monitoring focused more on worsening of skeletal abnormalities and functionality and did not include biochemistry. The panelists reached consensus on one statement regarding monitoring of disease progression in adult patients initiating enzyme replacement therapy: They recommended that these patients be seen every 3 months for the first year and then every 6–12 months thereafter to monitor disease progression, though it is worth noting that this might not be realistic for all patients, especially if they have to travel long distances to their centers with expertise. Therefore, it is reasonable to expect some variability in the intervals used in monitoring of disease progression, especially considering other factors (e.g., individual patient needs, complexity, heterogeneity of patients’ tolerability to medication, compliance with follow-up, other barriers to access). Interestingly, numerous areas in this domain failed to achieve consensus, such as the development of pseudogout, narcotic pain medication use, and worsening dental manifestations in the disease progression of adult patients with HPP. This could partly be explained by knowledge gaps in the research on how to evaluate/interpret changes over time within these manifestations in this specific population and thus would benefit from further research.

Panelists agreed on the role of centers with expertise and the need to refer patients when disease severity is not clear. The panelists also thought that a bone biopsy may offer information helpful to clinical management of adults with HPP, such as determining the degree of osteomalacia, which could be an indicator of disease severity. The SC agreed that while bone biopsy may have clinical utility, it may not be feasible in many centers and should not be required for all adults with HPP, as findings from a bone biopsy of a metabolic bone disease may differ between affected patients with HPP [36]. In certain patients with HPP who present with a complicated disease profile, bone biopsy may be indicated, and those patients should be managed in centers with expertise in managing patients with HPP where bone biopsy and histomorphometry services are available, when possible.

The SC also notes that it is important to acknowledge that HPP is an osteomalacia disorder that can coexist with osteoporosis; in fact, many patients attending osteoporosis clinics are clinically diagnosed with HPP when HPP is “unmasked” by treatment with bisphosphonates (worsening of signs/symptoms of HPP). Patients with HPP may also be misdiagnosed as having osteoporosis particularly if there is a strong history of fractures and pseudo-fractures. Molecular testing may assist with differential diagnosis in these cases [37].

Panelists agreed on statements highlighting the role of a multidisciplinary team in treating adult patients with HPP, which was also mentioned in the monitoring guidance for asfotase alfa-treated patients [5]. The panelists’ consensus on utilizing physical and occupational therapists when assessing disease severity is noteworthy as help from these providers is key to getting accurate assessments in real-world practice. The panelists agreed that some tests should be performed by a physical therapist, but others could be applied by the treating clinician in their regular clinical practice. While consensus was achieved on the role of physical/occupational therapists in providing physicians with objective data to assess disease severity, the SC acknowledges that these data are more commonly provided by physical therapists. A follow-up Delphi panel study including these physical and occupational therapists as panelists would provide additional insight into their role in the assessment of disease severity in adult patients with HPP.

Physicians’ understanding of how best to assess adult patients with HPP is evolving alongside our understanding of disease severity, and better assessment tools are being developed as our understanding of the ever-changing phenotype increases. Although the panelists agreed on the use of objective measures (e.g., hand grip strength), results from the EMPATHY study suggest that hand grip strength may not be a reliable tool to assess disease severity [38]; therefore, other measures (such as the 6MWT) may offer better methods to assess disease progression. In nonambulatory patients, physicians may need to consider other ways to assess disease severity.

Pain is a complex phenomenon and is highly subjective; therefore, using pain alone to assess disease progression may pose a challenge. Although consensus on using pain as a measure to assess disease progression in adult patients with HPP was not reached, the panelists agreed that pain assessment is a feasible and appropriate method to assess disease severity in the clinic. There was also consensus among panelists that there is a positive correlation between the amount of pain an adult patient with HPP reports and the severity of their disease. The panelists indicated that the types of specialist-prescribed pain medications the patient is taking are relevant to assessing disease severity; the frequency of pain medication use, while not mentioned in the consensus statements, may also be of interest. Increasing pain may be a symptom of progressive disease, so pain assessment may also be of benefit when assessing disease progression.

The results of this Delphi panel describe but do not provide definitions for disease severity or disease progression. There is a need for future research to develop definitions of mild, moderate, and severe disease for adult patients with HPP, which the panelists agreed would be useful in clinical practice. As a starting point, the SC proposes the following classifications for future research and debate: mild disease as patients who are able to walk at least 75% of the predicted distance on the 6MWT, moderate disease as those that fall between 35 and 75% of the percent predicted, and severe disease as patients who are nonambulatory or walk less than 35% of the 6MWT percent predicted. If it is not feasible to perform the 6MWT, using the TUG may prove beneficial in helping define disease severity. It has been shown that the TUG cutoff score for normal mobility in elderly patients is 10 to 12 s [26, 39], that completing the TUG in less than 20 s is correlated to independent functional transfers, and that taking longer than 30 s is correlated with being dependent for transfers, needing help to enter/exit the shower or tub and not going out of home alone [26]. Thus, mild disease might be defined as requiring less than 20 s to complete the TUG, moderate disease as requiring 20 to 30 s, and severe disease as patients who are nonambulatory or require longer than 30 s to complete the TUG. In addition to documenting the patient’s ability to complete the 6MWT and TUG Test, the SC would recommend documenting the patient’s use of ambulatory assistive devices (e.g., wheelchair, walker, cane, rollator) during the tests.

However, the SC acknowledges there are current limitations to this proposed classification. Defining severity based on the TUG or 6MWT does not account for the many nonspecific symptoms of HPP that can contribute to burden of disease in adults and thus may potentially result in clinicians determining a patient has “mild” disease when a broader view of the patient would be consistent with moderate disease. This publication may not be able to provide specific guidance on this, but we would anticipate this is a framework that will evolve over time. The panelists also agreed that substantial heterogeneity in the disease presentation across individuals must be considered and patients with HPP may experience changes in disease severity over time. In addition, the SC acknowledges that the TUG is not validated in HPP; it is suggested in the example classification because it is easier to incorporate in clinical practice than the 6MWT and is less of a burden on clinical staff. These example classifications are a starting point for discussions regarding monitoring in adult patients with HPP, and the TUG Test could be validated for this population in future research. Future research should also explore the use of other measures of functionality, such as the Lower Extremity Functional Scale or chair rise test, in this population. Finally, the SC suggests that including perspectives from other disciplines, such as physical therapy, in future discussions of HPP severity could lead to more well-rounded definitions.

Through the use of a Delphi panel, consensus was obtained on how to describe disease severity and disease progression in adult patients with HPP and how to monitor adults with HPP for disease progression in the clinical practice setting. This Delphi consensus is the first effort to try and describe these aspects of HPP. The statements regarding disease severity and assessment methods agreed upon by the expert panelists address some of the knowledge gaps in these areas and may be helpful for treating clinicians.

Limitations of the study include the small sample size and lack of sociodemographic and practice-related information (e.g., number of years managing adult patients living with HPP) about the panelists, limiting the understanding of the different experience/expertise of the sample. The sample was also limited to HCPs in the USA. Given the rare nature of the disease, panelists were recruited from the network of the SC, and it is thus possible they come from a similar school of thought. The response option “I don’t know,” as suggested by Vogel et al. 2019 [40], was not available in this study, meaning panelists were unable to indicate when they did not know the answer to a statement and could only select from the Likert options available. Including an “I don’t know” option would allow calculation only among participants who were confident in their answer. The “I don’t know” answers would then not be included in the calculation of consensus, whereas other responses (including neutral) would be included in calculations. Furthermore, due to the limited number of HCPs managing patients living with HPP, pilot testing was impractical. However, the 1:1 interviews used in round 1 ensured that any potential lack of clarity in the statements was rectified and/or missing discussion points were included in subsequent rounds. A further limitation is that the 80 statements added based on panelists’ comments during round 1 were only presented to the panelists in two rounds, as opposed to all three rounds.

To summarize, the consensus statements in this Delphi panel highlighted the role of fracture assessment, pain assessment, medication reconciliation, dynamic characterization of disease severity, and referral to appropriate rehabilitation specialists (physical therapy/occupational therapy). There was also consensus among panelists that there is a positive correlation between the amount of pain an adult patient with HPP reports and the severity of their disease. As our understanding of HPP continues to advance and as awareness of the disease increases, the results of this study may lay the foundation for integrating disease severity and progression into the nosology. These consensus statements may be useful in hypothesis generation for future research into clinical outcomes in patients with HPP.

### Supplementary Information

Below is the link to the electronic supplementary material.

Supplementary file1 (DOCX 69 KB)

## Data Availability

Some data generated or analyzed during this study are included in this published article or in the data repositories listed in References.
