# Supplementary material for: A Delphi panel to build consensus on assessing disease severity and disease progression in adult patients with hypophosphatasia in the United States
Source: J Endocrinol Invest. 2024 Jan 18;47(6):1487–97. doi: 10.1007/s40618-023-02256-4 (PMC11142979; doi:10.1007/s40618-023-02256-4)
Supplement: Supplementary file 1 — Supplementary file1 (DOCX 69 KB) [file 40618_2023_2256_MOESM1_ESM.docx]

**Supplemental Table 1. Summary of feasible and appropriate methods to assess disease severity in adult patients with HPP**

| **Treating physician** | **Physical Therapist/Occupational Therapist in lieu of the treating physician** |
| --- | --- |
| - Receiving objective data from Occupational Therapists and Physical Therapists (who are knowledgeable about HPP), providing valuable information with which to compare adult patients with HPP with controls on age and sex adjusted variables | - Assessments of muscle fatigue - Assessments of functional impairment using clinical scales (e.g. PROMIS, GMFM-88 and GSGC) - Five Times Sit to Stand Test - 6-Minute Walk Test - Timed Up and Go (TUG) Test - Quantifying the level of weakness displayed by the patient through objective methods such as hand grip strength or leg extensions |
| In clinic:   - Clinical consultations with the patient - Assessing quality of life via patient’s history and self-report - Assessments using pain scales (e.g., smiley faces, asking the patient to rate their level of pain on a 1-10 scale) - Assessing the types of pain medications the patient is taking, as prescribed by a specialist - Assessing the patient’s medical history - Checking for nephrocalcinosis - Assessing patient’s gait - Examining patient’s musculoskeletal condition   - Assessing the patient’s medical history   - Assessing their general ability to walk   - Performing X-rays to assess for fractures   - Assessing their gait |  |

Abbreviation: HPP, hypophosphatasia.

**Supplemental Table 2. Information on the 6MWT and TUG Test**

|  | **Six-Minute Walk Test^1^** | **Timed Up and Go Test^2^** |
| --- | --- | --- |
| **Description** | Patients are asked to walk back and forth as far as possible on a 30-meter walking course (i.e., 60-meter laps) for 6 minutes.  Patients should use their usual walking aids (e.g., cane or walker) and are permitted to slow down, stop, or rest as necessary. | Patients are asked to:   1. Stand up from sitting in a chair 2. Walk 3 meters 3. Turn 180 degrees 4. Walk 3 meters back to the chair 5. Sit down in the chair   Patients are cued to perform the test at a “comfortable and safe” pace.  No physical assistance is given, but patients may use their usual walking aids (eg, cane or walker). |
| **Score** | Distance travelled in 6 minutes (i.e., six-minute walking distance [6MWD])  Percent predicted values are also calculated, defined as the percent of normal predicted distance walked based on age, sex, and height, calculated as if the patient walked the full 6 minutes and is ≤65 years of age | Time taken, in seconds, to complete the test |
| **HPP validation reference** | Phillips et al 2018^3^ | Not applicable |
| **Proposed severity classifications^a^** | Mild: Ability to walk >75% of the predicted distance  Moderate: Ability to walk between 35%-75% of predicted distance  Severe: Non-ambulatory or ability to walk <35% of predicted distance | Mild: <20 seconds to complete  Moderate: 20-30 seconds to complete  Severe: Non-ambulatory or ≥30 seconds to complete |

^a^ Proposed classifications from Delphi panel steering committee for future research and debate.

Abbreviations: 6MWT, six-minute walk test; HPP, hypophosphatasia; TUG, Timed Up and Go.

**References**

1. ATS statement: guidelines for the six-minute walk test. Am J Respir Crit Care Med. 2002;166:111-7. doi:10.1164/ajrccm.166.1.at1102

2. Podsiadlo D, Richardson S. The Timed “Up & Go”: a test of basic functional mobility for frail elderly persons. J Amer Geriatr Soc. 1991;39:142-8. doi:/10.1111/j.1532-5415.1991.tb01616.x

3. Phillips D, Tomazos IC, Moseley S, L'Italien G, Gomes da Silva H, Lerma Lara S. Reliability and validity of the 6-minute walk test in hypophosphatasia. JBMR Plus. 2019;3:e10131. doi:10.1002/jbm4.10131
